# Efficacy of Anti-TNF Agents as Adjunctive Therapy for Knee Synovitis Refractory to Disease-Modifying Antirheumatic Drugs in Patients with Peripheral Spondyloarthritis

**DOI:** 10.1155/2013/907085

**Published:** 2013-06-11

**Authors:** Grigorios T. Sakellariou, Athanasios D. Anastasilakis, Ilias Bisbinas, Anastasios Gketsos, Charalampos Berberidis

**Affiliations:** ^1^Department of Rheumatology, 424 General Military Hospital, Thessaloniki, Greece; ^2^Department of Endocrinology, 424 General Military Hospital, Thessaloniki, Greece; ^3^Department of Orthopaedics, 424 General Military Hospital, Thessaloniki, Greece; ^4^Department of Orthopaedics, Bioclinic Hospital, Thessaloniki, Greece

## Abstract

Our aim was to evaluate the effectiveness of tumour necrosis factor (TNF) inhibitors as add-on therapy for knee synovitis that did not respond to disease-modifying antirheumatic drugs (DMARDs) and other standard treatments in patients with peripheral spondyloarthritis (SpA). We retrospectively studied 27 SpA patients, in whom an anti-TNF agent was added for active peripheral arthritis with knee synovitis refractory to DMARDs and treatment with low-dose oral corticosteroids and/or nonsteroidal anti-inflammatory drugs (NSAIDs) and intra-articular (IA) corticosteroids. As response of knee synovitis, were considered the absence of swelling, tenderness, and decreased range of movement in the clinical examination, after 4 months of anti-TNF therapy. In twenty-four (88.9%) of the patients there was response of knee synovitis. No statistical differences in gender (*P* = 0.53), age (*P* = 0.88), disease subtype (*P* = 0.22), and pattern of arthritis (*P* = 0.20) between knee synovitis responders and nonresponders were found. Fourteen patients managed to stop DMARD therapy and six, all of whom were initially on DMARDs combination, to decrease the number of DMARDs to one, maintaining simultaneously the response of knee synovitis. Our results imply a beneficial effect of adjunctive anti-TNF therapy on knee synovitis not responding to DMARDs and other standard treatments in patients with peripheral SpA.

## 1. Introduction

Diseases that belong to spondyloarthritides (SpA) are frequently manifested as asymmetric peripheral arthritis of the large joints with knee involvement. Tumour necrosis factor (TNF) inhibitors are highly effective for the treatment of peripheral arthritis in patients with ankylosing spondylitis (AS) [1, 2], psoriatic arthritis (PsA) [3], undifferentiated SpA (unSpA) [4], or SpA as a whole independently of the phenotypic disease [5], even in the case of arthritis resistant to disease-modifying antirheumatic drugs (DMARDs). However, data on the effect of anti-TNF therapy specifically on knee synovitis are limited in peripheral SpA [6]. The aim of this retrospective study was to evaluate the effectiveness of anti-TNF agents as adjunctive therapy for knee synovitis that did not respond to DMARDs and other standard treatments in patients with peripheral SpA.

## 2. Patients and Methods

We retrospectively studied patients with SpA according to the European SpA Study Group criteria [7] and peripheral arthritis involving the knee joint, who were monitored every 2–4 months at the rheumatology outpatient clinic of the 424 General Military Hospital (Thessaloniki, Greece) between January 2005 and January 2012. Inclusion criterion was the addition of an anti-TNF agent for active peripheral arthritis with knee synovitis unresponsive to DMARDs and standard treatment with low-dose oral corticosteroids (prednisone ≤7.5 mg/day) and/or nonsteroidal anti-inflammatory drugs (NSAIDs) and intra-articular (IA) corticosteroids. Exclusion criteria were (i) the use of IA corticosteroids and (ii) the increase of the DMARDs' dose or the addition of a new DMARD during anti-TNF therapy. Knee synovitis was defined as the presence of at least 2 of the following 3 clinical criteria: swelling, tenderness, or decreased range of movement. As response of knee synovitis the absence of the aforementioned three clinical joint signs after 4 months of anti-TNF therapy were considered. The association of knee synovitis response with gender, age, disease subtype, and pattern of arthritis at anti-TNF initiation excluding interphalangeal joint involvement was also investigated.

Knee synovitis responders and nonresponders were compared using the Mann-Whitney *U* test for continuous variables and the *χ* ^2^ test or Fischer's exact test for categorical variables. A *P* value of <0.05 was considered statistically significant in all tests. Statistical analysis was performed by using SPSS software for Windows, version 13 (SPSS Inc., Chicago, IL, USA).

## 3. Results

Twenty-seven patients with peripheral SpA were studied. Demographic and clinical characteristics of the patients are shown in Table 1. Twenty-one patients were on DMARD monotherapy (9 on methotrexate (MTX), eight on leflunomide (LEF), and four on sulfasalazine (SSz)) while the remaining 6 patients were on DMARDs combination treatment (2 patients on MTX + LEF, two on MTX + SSz, one on MTX + cyclosporine A (CysA), and one on MTX + CysA + LEF). Thirteen patients received adalimumab, 8 infliximab, 4 etanercept, and 2 golimumab. In all but three of the patients there was response of knee synovitis to anti-TNF therapy, which sustained for an average observation period of 31.9 months. Furthermore, all knee synovitis responders achieved at least low disease activity (LDA) according to the disease activity score in 28 joints (DAS28). Among the 3 patients with unresponsive knee synovitis, tenderness and decreased range of movement were the remaining signs in the two of them, who had psoriatic arthritis (PsA) with knee monoarthritis and achieved LDA. Knee joints of these two patients presented osteoarthritis grade 2 according to the Kellgren-Lawrence radiologic scoring system on X-ray, leading us not to consider treatment modification as appropriate approach, because the residual clinical signs of their knees were attributed to osteoarthritis secondary to joint destruction. In the third patient, who had oligoarticular PsA with remaining knee monoarthritis and achieved moderate disease activity (DAS28 = 3.29), both tenderness and swelling of the knee joint persisted. In this patient, whose knee X-ray was normal, a switch of adalimumab to infliximab was ineffective; finally, radiation synovectomy led to the resolution of knee synovitis. There were no differences in gender (*P* = 0.53), age (*P* = 0.88), disease subtype (*P* = 0.22), and pattern of arthritis (*P* = 0.20) between knee synovitis responders and nonresponders. All patients stopped NSAIDs and oral corticosteroids. Fourteen patients (51.9%) managed to stop DMARD therapy, while the rest 13 patients (48.1%) remained on combination of anti-TNF agent and DMARD in order to maintain both knee synovitis response and disease activity on remission or LDA; however, in 6 of these patients, all initially on DMARDs combination, the number of DMARDs was decreased to one.

## 4. Discussion

There is only one study in the literature evaluating the efficacy of anti-TNF agents focusing on knee synovitis [6]. In this prospective study, including 20 patients with peripheral SpA and knee synovitis who received etanercept as monotherapy, a statistically significant decrease in the physician's assessment of the knee joint (median visual analog score from 53.5 at baseline to <10 at week 12 and thereafter) was observed. Accordingly, clinical synovitis of the knee joint was present in all patients at baseline and decreased to a frequency of 12 (60%) of the 20 knee joints with synovitis at week 12 and 6 (30%) at week 52. In comparison, knee synovitis persisted in approximately 11% of our SpA patients at month 4 following anti-TNF addition. The combination of anti-TNF agent with DMARDs could explain the more favorable results of our study. Indeed, at least in regard to PsA, the superiority of anti-TNF agent plus MTX to MTX alone is well documented in MTX-naïve patients [8] and, according to the Danish registry, concomitant use of DMARD (MTX) is a predictor of clinical response in patients treated with an anti-TNF agent [9]. Furthermore, it has been reported that in PsA patients, early treatment before joint damage resulted in better outcome and polyarticular disease had poorer prognosis than oligoarticular disease [10, 11]. Thus, the addition of an anti-TNF agent shortly after DMARD failure (mean time interval between initiation of DMARDs and anti-TNF agent was 11.4 months, Table 1) and the fact that none of our patients with PsA had polyarticular disease could be additional reasons for the better response of knee synovitis in our study. Notably, the two PsA patients with radiographic damage of the affected knee joint at baseline were nonresponders to anti-TNF therapy. In conclusion, the high percentage of our SpA patients with knee synovitis responsive to anti-TNF agent may be explained by the combination of anti-TNF therapy with DMARDs and the presence of good response predictors in most of them, although in the literature this could be supported only for the PsA patients, who were the majority of the studied patients.

Our study has several limitations. It is a retrospective study with limited number of patients, who had heterogeneity on pattern of DMARD therapy and were treated with different anti-TNF agents and DMARDs. Furthermore, we did not include a control group. However, this is the first study to attempt to evaluate the efficacy of anti-TNF agents as adjunctive therapy for knee synovitis refractory to DMARDs in patients with peripheral SpA. In all patients, knee synovitis had already failed to respond to DMARDs and other standard treatments before the addition of an anti-TNF agent. Thus, it is reasonable to attribute knee synovitis response to anti-TNF therapy itself. The absence of swelling, tenderness, and decreased range of movement on clinical examination, which has been used as outcome in randomized controlled studies [12, 13], was considered as response of knee synovitis in our study. We used the term “response” over “remission” in order to exclude the possibility of mild knee joint inflammation not detectable with clinical examination.

## 5. Conclusion

Our results imply a sustained beneficial effect of adjunctive anti-TNF therapy on knee synovitis unresponsive to DMARDs and other standard treatments in patients with peripheral SpA. Because of both knee synovitis response and remission or LDA, approximately half of the patients stopped DMARDs and all patients on DMARDs combination decreased the number of them to one. However, large prospective studies are required to confirm our results.

## Figures and Tables

**Table 1:** Demographic and clinical characteristics of the studied patients.

Parameter	All patients (*n* = 27)	Knee synovitis responders (*n* = 24)	Knee synovitis nonresponders (*n* = 3)
Age (years)	43.3 ± 2.9	43.1 ± 3.2	44.7 ± 1.3
Age at disease onset (years)	36.0 ± 2.6	36.7 ± 2.8	30.7 ± 1.3
Time interval between initiation of DMARDs and anti-TNF agent (months)	11.4 ± 1.1	11.3 ± 1.3	11.9 ± 0.5
Male gender	19 (70.4)	16 (66.6)	3 (100.0)
Disease subtype			
AS	2 (7.4)	2 (8.3)	0 (0.0)
PsA	17 (63.0)	14 (58.3)	3 (100.0)
unSpA	8 (29.6)	8 (33.3)	0 (0.0)
HLA-B27*			
Positive	4 (66.6)	4 (66.6)	—
Negative	2 (33.4)	2 (33.4)	—
Pattern of DMARD therapy before addition of anti-TNF agent			
Monotherapy	21 (77.8)	18 (75.0)	3 (100.0)
Combination	6 (22.2)	6 (25.0)	0 (0.0)
Pattern of arthritis on anti-TNF initiation			
Monoarthritis	8 (29.6)	6 (25.0)	2 (66.6)
Oligoarthritis (inflamed joints <4)	19 (70.4)	18 (75.0)	1 (33.4)
Current therapy			
Anti-TNF monotherapy	14 (51.9)	12 (50.0)	2 (66.6)
Anti-TNF + DMARD	13 (48.1)	12 (50.0)	1 (33.4)

DMARDs: disease-modifying antirheumatic drugs; TNF: tumour necrosis factor; AS: ankylosing spondylitis; PsA: psoriatic arthritis; unSpA: undifferentiated spondyloarthritis.

*Included only the patients with known HLA-B27 status (*n* = 6).

Values given as mean ± standard error of the mean (SEM) or *n* (%).
